# Identification and Structural Characterization of a Novel *COL3A1* Gene Duplication in a Family With Vascular Ehlers–Danlos Syndrome

**DOI:** 10.1002/mgg3.70095

**Published:** 2025-04-12

**Authors:** Gianmaria Miolo, Piernicola Machin, Marco De Conto, Sara Fortuna, Simona Viglio, Lara Della Puppa, Silvano Geremia, Giuseppe Corona

**Affiliations:** ^1^ Medical Oncology and Cancer Prevention Unit Centro di Riferimento Oncologico di Aviano (CRO), IRCCS Aviano Italy; ^2^ Pathology Unit, Department of Medicine Laboratory Section Pordenone Hospital Pordenone Italy; ^3^ Department of Chemical and Pharmaceutical Sciences, Centre of Excellence in Biocrystallography University of Trieste Trieste Italy; ^4^ Department of Molecular Medicine, Biochemistry Unit University of Pavia Pavia Italy; ^5^ Oncogenetics and Functional Oncogenomics Unit Centro di Riferimento Oncologico di Aviano (CRO), IRCCS Aviano Italy; ^6^ Immunopathology and Cancer Biomarkers Unit Centro di Riferimento Oncologico di Aviano (CRO), IRCCS Aviano Italy

**Keywords:** *COL3A1* gene, collagen, duplication, genetic variant, metalloproteinase, vascular Ehlers–Danlos syndrome

## Abstract

**Background:**

Vascular Ehlers–Danlos syndrome (vEDS) is caused by alterations in the *COL3A1* gene, typically involving missense variants that replace glycine residues. In contrast, short *in‐frame* insertions, deletions, and duplications are rare and pose significant challenges for investigation.

**Methods:**

The histological examination of vascular tissue from a 26‐year‐old man, who died from a common iliac artery aneurysm and whose mother died at age 60 from an abdominal aortic dissection, strongly suggested a diagnosis of Ehler–Danlos type IV. Ex vivo collagen phenotype assessment, molecular analysis, and in silico structural studies of type III collagen were subsequently performed.

**Results:**

Ex vivo analysis of the patient's fibroblasts revealed altered collagen synthesis, whereas the molecular testing identified a novel 18‐nucleotide *in‐frame* duplication (c.2868_2885dup‐GGGTCTTGCAGGACCACC) in the *COL3A1* gene, resulting in a six‐amino acid insertion, p.(Leu958_Gly963dup). Structural investigation indicated that this duplication led to a local perturbation of the collagen triple helix near a metalloproteinase cleavage site.

**Conclusion:**

This study highlights the pathogenic role of a novel *in‐frame* duplication in the *COL3A1* gene, demonstrating how this seemingly benign alteration significantly compromises collagen turnover and contributes to the development of vEDS.

## Graphical abstract

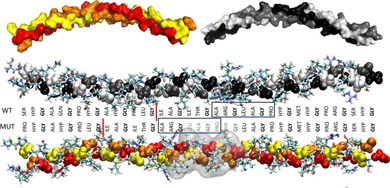

## 1 Introduction

Ehlers–Danlos syndrome (EDS) encompasses a genetically heterogeneous group of connective tissue disorders, each resulting from distinct alterations in collagen genes (Chiarelli et al. 2019).

Among all collagens, type III is the second most abundant fibrillar collagen in the body, frequently combining with other collagens to form a strong fibrous network that provides essential structural support to various tissues such as skin, blood vessels, and muscles (Wang et al. 2020; Omar et al. 2021).

The mechanical stability, elasticity, and strength of type III collagen are maintained through a series of intricate processes that create a stable, twisted, rope‐like structure. Proper formation of the triple‐helix structure, characteristic of all collagen isoforms, depends on the repeated sequence: Gly‐Xaa‐Yaa, where the glycine (Gly) residue is often followed by imino acid residues, proline (Pro) in Xaa positions, and hydroxyproline (Hyp) in Yaa positions (Taga et al. 2021). Due to its small size, the Gly amino acid plays a key role in fitting within the narrow internal space of the triple helix, ensuring the correct packing of the three chains (Omar et al. 2021). In this context, the replacement of a Gly residue with any other amino acid leads to the formation of an abnormal triple‐helix structure due to the bulkier side chain.

Pathogenic variants in the *COL3A1* gene are responsible for vascular Ehlers–Danlos syndrome (vEDS) (#130050), a condition typically characterized by the fragility of blood vessels, making individuals highly susceptible to arterial ruptures and aortic aneurysms (Byers et al. 2017).

The majority of these pathogenic variants are heterozygous missense substitutions that specifically target Gly residues within the 343 [Gly‐Xaa‐Yaa] repeats of the triple helical region, leading to the production of altered proteins which negatively affect the activity of the non‐mutated allele product, disrupting the stable assembly of type III procollagen homotrimers (dominant‐negative effect) (Pepin et al. 2014; Leistritz et al. 2011; Frank et al. 2015; Yagi et al. 2022). Less commonly observed are frameshift, nonsense, or large deletion variants, which lead to a 50% reduction in mature collagen levels, thereby affecting the physiological function of type III collagen (haploinsufficiency) (Schwarze et al. 2001). Interestingly, these latter variants result in milder phenotypes, while those causing the formation of abnormal proteins are associated with more severe phenotypes (Ghali et al. 2019).

Instead, the short *in‐frame* insertions, deletions, and duplications of the *COL3A1* gene are rarer, making it challenging to investigate their precise and effective pathogenic role in determining vEDS.

This study, for the first time, reports a *COL3A1* gene duplication involving 18 base pairs corresponding to an *in‐frame* sequence of six amino acids (Leu‐Ala‐Gly‐Pro‐Pro‐Gly) associated with a phenotype highly suggestive of vEDS. Clinical, histological, phenotypic, molecular, and structural characterization of the index case supports the pathogenic role of this gene variant, which locally alters the triple‐helix structure of collagen and may affect metalloproteinase activity impacting the COL3A1 turnover.

## 2 Materials and Methods

### 2.1 Ethical Compliance

This study was conducted in accordance with the ethical standards outlined in the 1964 Declaration of Helsinki and its subsequent amendments. All the patients provided informed consent for the execution of molecular investigations and the publication of research findings. Patients' personal data were fully anonymized in accordance with the General Data Protection Regulation (GDPR). Formal ethical committee approval was not required.

### 2.2 Study Participants

The study focused on a family whose members exhibited clinical signs suggestive of vEDS. The index case underwent surgery at the age of 15 for a pseudoaneurysm of the left popliteal artery, initially suspected to be of post‐traumatic origin. At 22 years old, the proband experienced a frontotemporal ischemic stroke resulting from occlusive dissection of the left internal carotid artery, which led to residual right hemiparesis and primarily motor aphasia. Afterwards, the proband was hospitalized for hemorrhagic shock due to the rupture of an aneurysm in the left distal common iliac artery, near its bifurcation. A few days later, the patient developed ischemic colitis accompanied by rectal bleeding requiring further hospitalization. A CT scan revealed active peri‐cholecystic bleeding, which required embolization of the proper hepatic artery. After the procedure, the patient experienced a cardiocirculatory arrest that led to his death at the age of 26. Analogously, his mother had died at the age of 60 for vascular complications arising from abdominal aortic dissection.

### 2.3 Histological and Immunohistochemical Examination

Blood vessel samples underwent initial fixation in 10% neutral buffered formalin followed by embedding in paraffin to create tissue blocks. These blocks were then sectioned into 4 μm slices. After deparaffinization, the sections were prepared for staining. For a comprehensive assessment of basic tissue morphology, Hematoxylin and Eosin (H&E) staining was applied to the deparaffinized section, facilitating clear visualization of cellular structure and tissue architecture.

To pinpoint specific constituents within endothelial cells and the basement membrane, the periodic acid‐Schiff with diastase (PASD) digestion method was employed. This technique enables the selective detection of glycogen and other polysaccharides. Additionally, the smooth muscle actin (SMA) staining technique was utilized to identify the smooth muscle cells within the tissue. Silver staining was applied to visualize the intricate structure of the blood vessels, particularly focusing on the collagenous components of the vessel walls.

### 2.4 Ex Vivo Collagen Synthesis Analysis

Fibroblast cultures, established from a skin biopsy of the proband, were grown and maintained in Dulbecco modified Eagle's medium (DMEM) supplemented with 10% fetal calf serum. Metabolic labeling of cells was carried out using a serum‐free medium containing 50 μg/mL ascorbate and 30 μCi of ^3^H‐labeled proline (L‐[2,3‐^3^H]proline). Type I, III, and V collagens were subsequently purified from both the medium and cell layer as previously described (Valli et al. 1991) and then separated via 6% sodium dodecylsulfate–polyacrylamide gel electrophoresis (SDS–PAGE) in the presence of urea. Gel processing for fluorography was conducted using standard procedures, and quantitation was performed using the Image J program released by the National Institute of Health, USA. The range of values for the normal type III/I collagen ratio was determined by measuring this ratio in various normal subjects over a period of 3 years.

### 2.5 Molecular Genetics Analysis

Exome sequencing (ES) was conducted on DNA extracted from the proband's whole blood using a column‐based extraction kit according to the manufacturer's instructions (NucleoSpin Blood DNA; Macherey‐Nagel, Düren, Germany). ES of the proband utilized the Agilent SureSelect All Exon V6 kits for exome enrichment and sequencing was performed on a NovaSeq6000 platform (Illumina). Reads were aligned to the human reference genome (UCSC hg19) using Burrows–Wheeler Aligner (BWA, V.0.7.8‐r455). Over 99% of the targeted regions were sequenced to > 20× coverage. High‐quality indel and single nucleotide variant calling and annotation were carried out using GATK v3.1 and SAMtools v.0.1.7 with standard filtering criteria. Copy number variations (CNVs) were detected using ExomeDepth and Pindel.

Variants were prioritized based on a minor allele frequency (MAF) < 1% and cross‐referenced in public databases including gnomAD Exomes, gnomAD Genomes, ClinVar, Leiden Open Variant Database (LOVD) and Human Gene Mutation Database (HGMD). The reference sequence for the *COL3A1* gene used in this study was NM_000090.3 from GenBank. The variants underwent classification according to the American College of Medical Genetics (ACMG) guidelines (Richards et al. 2015). Any variants deemed pathogenic, likely pathogenic, or of unknown significance were selected for further validation through Sanger sequencing.

### 2.6 Structural Analysis of *COL3A1* Variant

The set of collagen trimeric fibrils has been built by taking the crystallographic structure with PDB code 1BKV as a template (Kramer et al. 1999). Each fibril was placed in a cubic box with a water layer of minimum of 1.0 nm and neutralized with Cl^−^ ions. The AMBER99SB‐ILDN force field and tip3p water model were used. Steepest descent minimizations of the protein chains were stopped either when the maximum force was lower than 1000.0 kJ/mol/nm or when 50,000 minimization steps were performed with a 0.005 kJ/mol energy step size, with a Verlet cutoff scheme, short‐range electrostatic cut‐off, and Van der Waals cut‐off of 1.0 nm. We performed NVT (constant Number of particles, Volume, and Temperature) and NPT (constant Number of particles, Pressure, and Temperature) equilibrations for 100 ps, constraining the protein backbone. NPT simulations were run both at 300 K and 320 K (modified Berendsen thermostat) at 1 atm (Parinello‐Rahman pressure coupling) for 500 ns. The iteration time step was set to 2 fs with the leap‐frog integrator and LINCS (Hess et al. 1997) constraint with periodic boundary conditions. All the simulations and their analysis were run as implemented in the Gromacs package v. 2021.4 (Pronk et al. 2013). The binding free energy was estimated with the MM/GBSA method over 500 configurations sampled every 1 ns, with the apolar solvation energy calculated as solvent accessible surface area and default parameters, as implemented in the gmx Molecular Mechanics Poisson–Boltzmann Surface Area (MMPBSA) tool (Valdés‐Tresanco et al. 2021). The trimeric polypeptides’ end‐to‐end distances (L) with their standard deviations (s) were monitored, considering the measurement between the third and last third C‐alpha atoms of the middle B chain to avoid issues related to partial unfolding of the terminals. The curvature (1/*r*) of the trimeric peptide was monitored throughout the simulation. This was done by calculating the best circle passing through the C‐alpha atoms, omitting the first three and last three amino acids of the leading A chain, the first two and last two amino acids of the middle B chain, and the first and last three amino acids of the trailing C chain. The algorithm uses singular value decomposition (SVD) to find the best fitting plane to project the 3D points into 2D space and the least‐squares method to fit a circle with radius r to the 2D coordinates. The flexibility (*f*) of trimeric peptides was calculated as the s of the time‐dependent curvature. The root mean square deviation (RMSD) which measures the collective displacement of the backbone atoms over time, the root mean square fluctuation (RMSF) which provides a time‐averaged measure of the displacement of each backbone atom L, and curvatures were calculated from configurations sampled every 500 ps. Five sets of sampled configurations, each set shifted by 100 ps, were used to calculate the L and curvatures, estimating the standard error. Representative conformations were generated by clustering simulations snapshots with the Daura algorithm (Daura and Conchillo‐Solé 2022), cutoff 0.25 nm, as implemented in GROMACS. Simulations were run on M100 (CINECA, Italy).

## 3 Results

**FIGURE 1:**
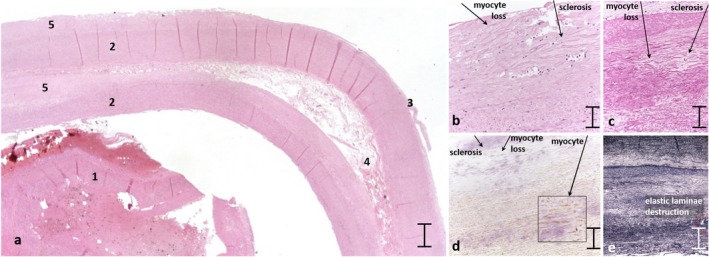
(a) (2.5×, scale bar = 500 μm): (1) thrombus within the vascular lumen; (2) media layer; (3) adventitia layer; (4) dissecting hematoma within the media layer; (5) wall degeneration. Figure 1b–e: (20×, scale bar = 100 μm) Four specific stains are displayed: (b) H&E, (c) PASD, (d) SMA, and (e) Silver. The H&E, PASD, and SMA staining reveals an evident wall degeneration characterized by myocyte loss and sclerosis, whereas the silver staining highlights the elastic laminae destruction.

Arterial vessel tissue examination from the index case through H&E staining reveals a dissecting hematoma within the artery's media layer, compromising the integrity of the vessel wall (Figure 1). To enhance the detection of vessel alterations, additional standard staining techniques including PASD and SMA were employed alongside H&E. These methods collectively highlighted the loss of myocytes and the concurrent presence of sclerosis (Figure 1b–d). The SMA staining, which targets SMA, provided enhanced visualization of the smooth muscle cells distribution within the vessel tissue, indicating valuable insights into abnormalities of the smooth muscle composition and arrangement (Figure 1d). Furthermore, Silver staining application showed a clear destruction of the elastic laminae (Figure 1e), contributing to additional details in support of the structural changes in the vessel wall.

**FIGURE 2:**
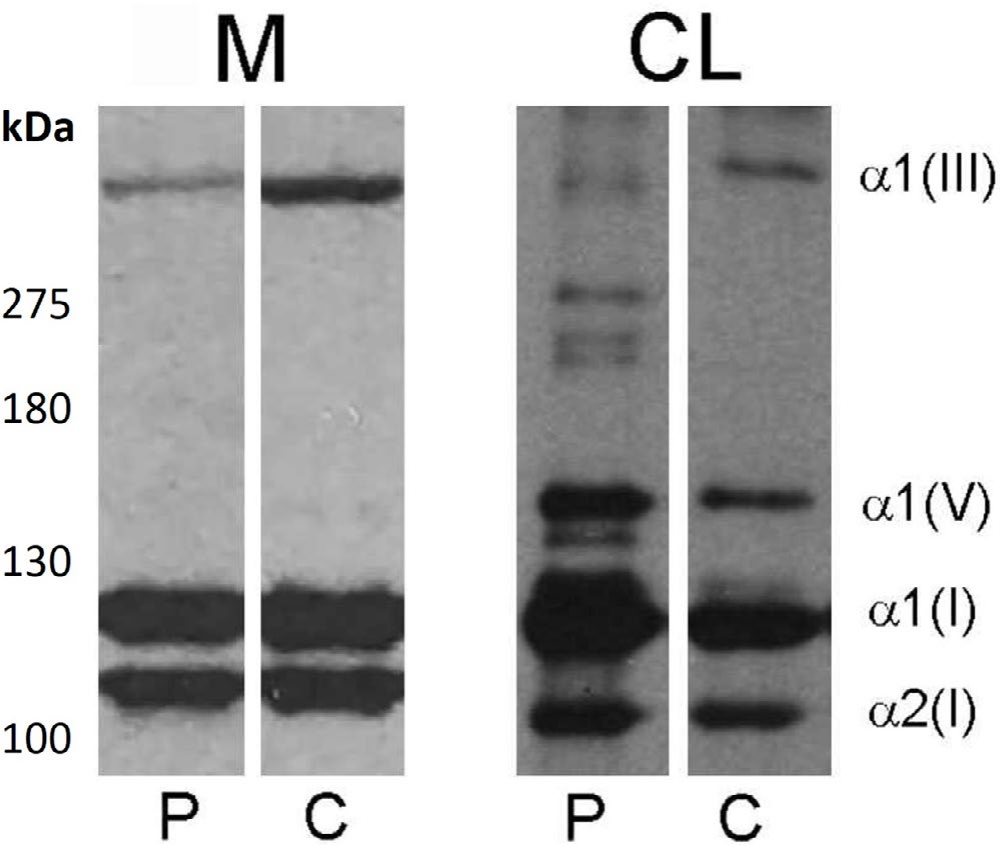
6% SDS–PAGE of pepsin‐treated collagen secreted by fibroblasts (M) and retained in cell layer (CL) of a patient affected by vEDS (P) and an age‐matched control (C).

Collagen profile synthesis from fluorography SDS–PAGE analysis revealed that in the cell layer (CL) the type III collagen chains displayed normal electrophoretic migration but exhibited lower cellular accumulation compared to the control. Moreover, in the CL sample of the proband, distinct bands were observed between the molecular weights of the collagen chains III and I, potentially indicating degradation products of collagen III. This observation was consistent with the levels of collagen III released in the cell medium (M), which were significantly lower compared to the control. The signal of the type III/I collagen ratio was also lower in the proband, suggesting a qualitative defect in the stable form of type III collagen (Figure 2).

**FIGURE 3:**
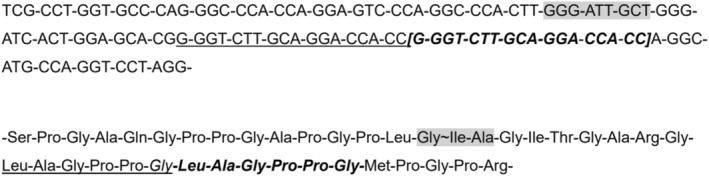
The location of the 18 nucleotides duplication in the COL3A1 gene and the corresponding insertion of 6 amino acids into the type III α1 chain are underscored. The MMP cleavage site, shown both as nucleotide and amino acid sequence, is highlighted in grey.

Molecular analysis of the *COL3A1* gene performed in the proband highlighted a heterozygous duplication of 18 nucleotides NM_000090.3: c.2868_2885dup‐GGGTCTTGCAGGACCACC resulting in an *in‐frame* duplication characterized by the insertion of 6 amino acids, p.(Leu958_Gly963dup) (Figure 3) that has not been previously reported in population databases (gnomAD) or in mutational databases (LOVD, HGMD). The same heterozygous genetic rearrangement was also detected in his 22‐year‐old proband's brother who currently does not show clinical manifestations, while it was not present in the proband ‘s father.

**FIGURE 4:**
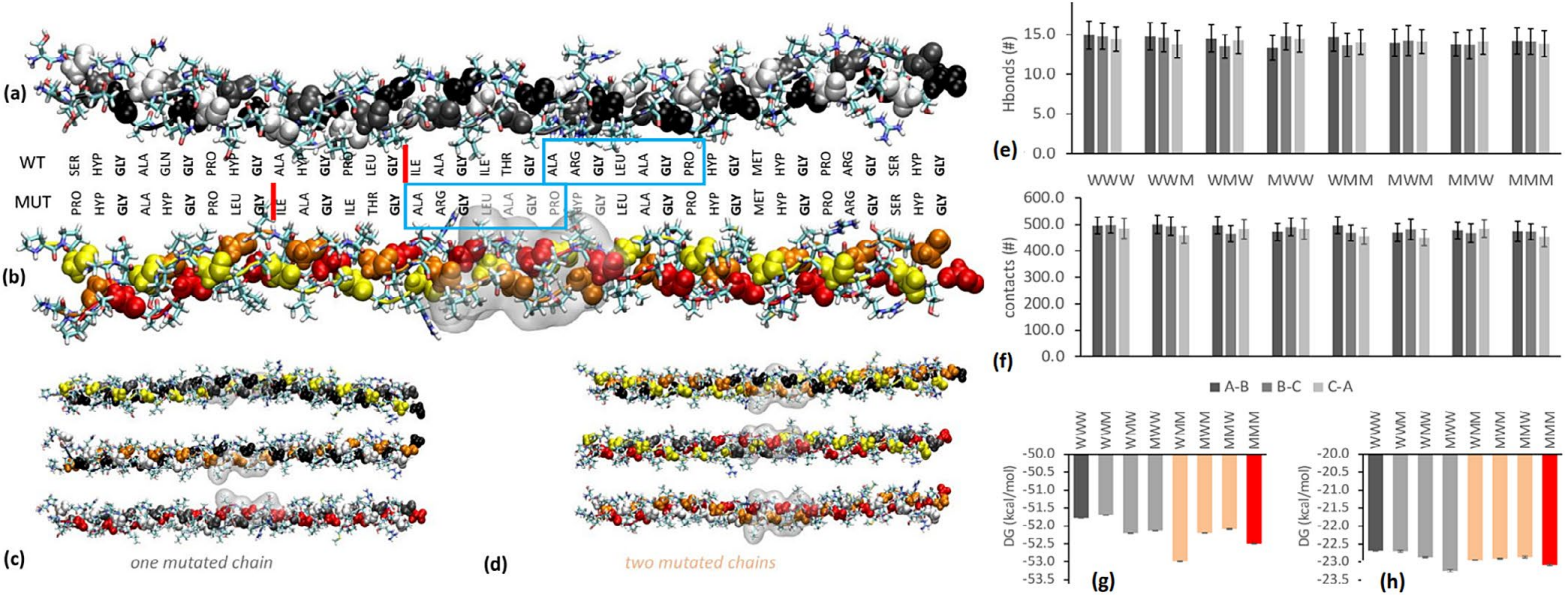
Representative conformations: (a) wild type; (b) mutated homotrimer; (c) hetero‐trimers with one mutated chain; (d) heterotrimers with two mutated chains. Colour code: Wild type chain leading A (white), middle B (grey), trailing C (black); mutant chain leading A (yellow), middle B (orange), trailing C (red). In all systems Pro residues are highlighted by van der Waals spheres. Insertions are highlighted as well as transparent grey surfaces. The unique collagenase cleavage site between the Gly and Ile residues is marked with a red bar. The exosite recognized by the hemopexin domain of MMP‐1, crucial for cleaving the collagen triple helix, is highlighted with a blue box and it overlaps with the mutated region. Trajectories analysis: (e) number of interchain hydrogen bonds and (f) number of contacts, average over 500 ns of molecular dynamics simulation; (g) average binding free energy be‐tween two chains and (h) between one chain and the other two. Energies were calculated over 500 ns of molecular dynamics simulation with the MM/GBSA method. Error bars are standard deviations.

**FIGURE 5:**
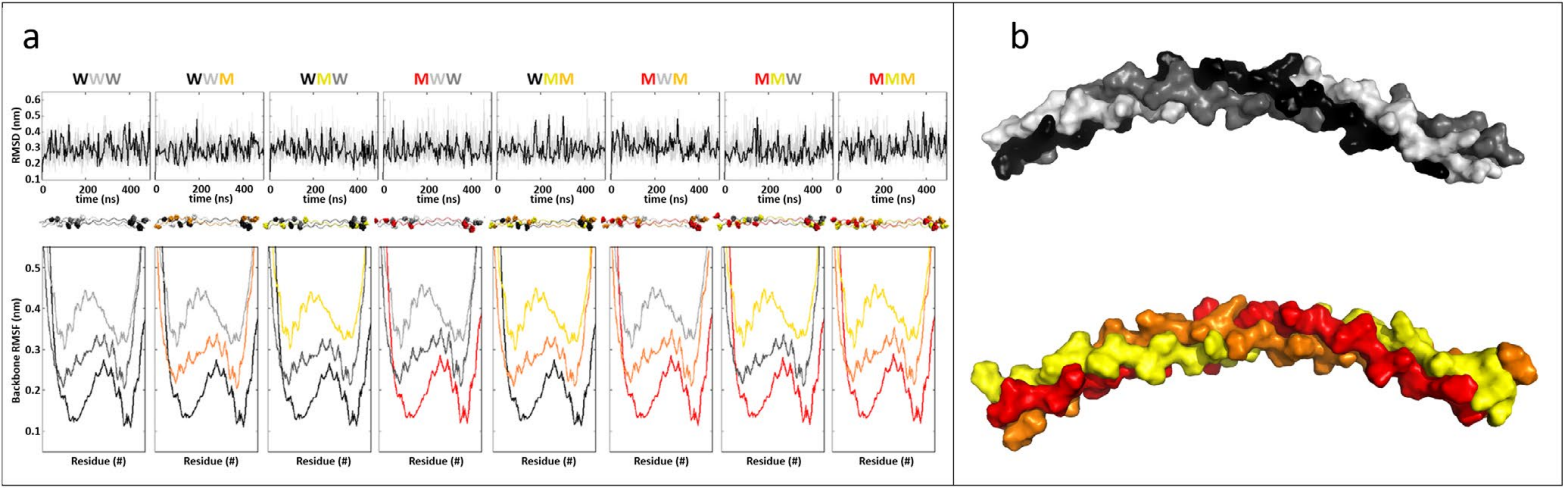
Molecular dynamics results. (a) For each model: Protein backbone root mean squared deviation (RMSD) in grey the original dataset, in black the same data spline‐smoothed; 500 ns trimers conformation with the Hyp groups highlighted; and root mean squared fluctuation (RMSF) for each chain, where the curves of chain C and B have been displaced by 0.1 and 0.2 nm, respectively. Color code: Wild type (black/white‐grey/grey), mutants (red/yellow/orange). (b) Snapshots corresponding to the maximum deformation of the collagen observed during the simulation: 0.0104 nm^−1^ for the wild type, and 0.0116 nm^−1^ for the mutated homotrimer, respectively. Color code: Wild type (black/grey/white), mutant (red/orange/yellow). The movies with the entire dynamics are available as Supporting Information.

In order to understand the structural effects of the p.(Leu958_Gly963dup) duplication, a set of in silico trimeric collagen models was built and energetic stability investigations have been performed. Indeed, the *COL3A1* gene encodes the collagen α‐1(III) chain, known to assemble into the triple‐helical structure characterizing type III collagen (Kuivaniemi and Tromp 2019). Using the crystal structure of a known collagen peptide model as a template (Kramer et al. 1999) a 39 amino acid peptide for both the wild type and mutant homotrimers was constructed, placing the duplicate sequence in the middle of the model. Each modeled chain comprises 13 (Gly‐Xaa‐Yaa‐) triplets and includes a total of six hydroxyproline residues (Figure 4). This composition, which supports the formation of the canonical triple‐helical structure of collagen (Jalan et al. 2020; Boudko et al. 2008) generates eight heterotrimer models when the duplication variant is considered. Each model was achieved by aligning all chains at the C‐terminus region, which is crucial for type III collagen assembly. The general pattern (−Gly‐Xaa‐Yaa‐), with a Gly every third residue, was conserved in all eight mutant models (Figure 4a–d). Throughout the simulation, all models tended to maintain their overall conformation, as confirmed by measuring the trimer's backbone RMSD and each chain's RMSF. These mobility parameters remained consistently stable, with values below 0.6 nm, and only minor differences were observed across the models (Figure 5). Specifically, the RMSF graphs displayed the expected maximum displacement in the central imino‐poor region, as well as at the N‐terminal and C‐terminal ends of the polypeptides. Additionally, the hydrogen bonds formed by the hydroxyl groups of hydroxyprolines, which are crucial for maintaining the thermal stability of the collagen triple helix structure (Matthew et al. 2009) were consistent across all models, with approximately 14 hydrogen bonds observed in each case (Figure 4e).

A similar observation was made regarding the number of contacts among chains. There was a slight reduction in the number of contacts estimated by MMPBSA as the number of mutated chains increased in the model (Figure 4f). A decrease in the number of contacts can suggest a possible weakening of interchain interactions as a function of the increasing number of mutated chains; however, without significantly affecting thermodynamics. Indeed, the difference in energy required to separate a single chain from the trimer was within 1.3 kcal/mol for all cases (Figure 4g), while the energy needed to separate two chains was within 0.6 kcal/mol (Figure 4h). Therefore, within the reliability of these calculations, the duplication does not appear to significantly affect the thermodynamic stability of the triple‐helical structure.

To investigate the effect of mutation on the flexibility of the triple helix, the *L* of the trimeric polypeptides was monitored during the dynamic simulations (Figure 5). The wild‐type helix exhibited an average *L* of 10.147 ± 0.007 nm with a s of 0.102 ± 0.008 nm. In contrast, the mutated homotrimer displayed a slightly shorter average *L* of 10.123 ± 0.003 nm with a higher s of 0.118 ± 0.007 nm. This trend was further corroborated by evaluating the mean curvature (1/*r*) and the corresponding flexibility (*f*), calculated as the s of the time‐dependent curvature of the trimeric helix. The calculated 1/*r* values were 0.00329 ± 0.00009 nm^−1^ for the wild type and 0.0355 ± 0.00010 nm^−1^ for the mutated homotrimer. Correspondingly, the flexibility (*f*) values were 0.00184 ± 0.00013 nm^−1^ and 0.00203 ± 0.00013 nm^−1^ for the wild type and mutated homotrimer, respectively.

## 4 Discussion

Type III collagen is a triple helix protein comprising three α1 amino acid chains intertwined through trimeric interactions, which are crucial for the stability and strength of collagen. Any perturbation of this structure typically results in the weakening or instability of collagen, leading to an increased risk of arterial rupture and dissection (Kuivaniemi and Tromp 2019; Boudko et al. 2008).

In the present investigation, the clinical and the histological features revealed in the index case and his mother, both of whom died from aneurysm development and vessel rupture, strongly suggested alterations in the structural integrity of the vessel wall. The sclerosis and myocellular loss of the tunica media, coupled with the destruction of the elastic laminae (Figure 1a–e) provided compelling evidence for considering a potential diagnosis of vEDS. Sequencing of the *COL3A1* gene in the proband highlighted a heterozygous duplication of 18 nucleotides NM_000090.3: c.2868_2885dup located in exon 40. This variant is characterized by an *in‐frame* duplication of 6 amino acids (Leu‐Ala‐Gly‐Pro‐Pro‐Gly), p.(Leu958_Gly963dup), which preserves the common collagen triplet repeat sequence motif. The identification of this variant in both the index case and his brother, combined with its absence in the father, indicated a potential maternal transmission. This specific variant appears to be novel, being not registered in the dbSNP and gnomAD *databases* and, according to the American College of Medical Genetics (ACMG) guidelines, can be classified as a pathogenic variant (PVS1 and PM2 criteria) (Richards et al. 2015).

*In‐frame* duplications of the *COL3A1* gene occurring in multiples of three amino acids have been rarely reported. Among these, the variant c.2299_2316dup, characterized by the *in‐frame* insertion of the Ile‐Gly‐Pro‐Pro‐Gly‐Pro amino acid sequence, was associated with an atypical mild phenotype characterized by the sudden onset of consciousness disorders and abdominal pain, alongside multiple intra‐abdominal aneurysms (Xu et al. 2019) while the c.3124_3141dup p.(Ala1042_Gly1047dup) duplication, resulting in the insertion of the Ala‐Pro‐Gly‐Ala‐Pro‐Gly amino acid sequence, was found to co‐segregate with the vEDS phenotype within a large family (Hayashi et al. 2022). Additionally, the duplication c.3441_3485dup, leading to the *in‐frame* insertion of 15 amino acids Lys‐Asp‐Gly‐Thr‐Ser‐Gly‐His‐Pro‐Gly‐Pro‐Ile‐Gly‐Pro‐Pro‐Gly, was detected in a 42‐year‐old female exhibiting spontaneous carotid‐cavernous fistula, a hallmark of vEDS (Frank et al. 2015; Kiss et al. 2018). Similarly, other *in‐frame* duplications such as the c.615_629dup p.(Gly207_Pro211dup) and the c.3221_3235dup p.(Gly1074_Pro1078dup), characterized by the insertion of five amino acids, have been strongly associated with the vEDS phenotype (Yang et al. 2007; Pepin et al. 2014; Adham et al. 2018) (Table 1). Interestingly, all these variants have been identified in families where there was a strong clinical suspicion of vEDS.

**TABLE 1:** In‐frame duplication alterations in the *COL3A1* gene.

DNA change (cDNA)	Ex	Protein	AAs duplication	Outcome	ACMG classification	Criteria	References
c.2299_2316dup	33	p.(Ile767_Pro772dup)	6 (IGPPGP)	In‐frame dup	Likely pathogenetic score 9	PVS1, PM2, BP3	Hayashi et al. (2022)
c.3124_3141dup	43	p.(Ala1042_Gly1047dup)	6 (APGAPG)	In‐frame dup	Pathogenetic score 10	PVS1, PM2	Kiss et al. (2018)
c.3441_3485dup	47	p.(Lys1150_Gly1164dup)	15 (KDGTSGHPGPIGPPG)	In‐frame dup	Pathogenetic score 10	PVS1, PM2	Adham et al. (2018)
c.615_629dup	7	p.(Gly207_Pro211dup)	5 (GQAGP)	In‐frame dup	Pathogenetic score 10	PVS1, PM2, PP5, BP3	Pepin et al. (2014)
c.3221_3235dup	44	p.(Gly1074_Pro1078dup)	5 (GAPGP)	In‐frame dup	Pathogenetic score 10	PVS1, PM2	Yang et al. (2007)
c.2868_2885dup	40	p.(Leu958_Gly963dup)	6 (LAGPPG)	In‐frame dup	Pathogenetic score 10	PVS1, PM2	Present study

Abbreviations: AAs, amino acids; ACGM, American Collage Medical Genetics; BP3, in‐frame deletions/insertions in a repetitive region without a known function; Caveat, population data for insertions/deletions may be poorly called by next‐generation sequencing; Ex, exon; PM2, absent from controls (or at extremely low frequency if recessive) in Exome Sequencing Project 1000 Genomes Project or Exome Aggregation Consortium; PP5, reputable source recently reports variant as pathogenic, but the evidence is not available to the laboratory to perform an independent evaluation; PVS1, null variant (nonsense frameshift canonical ±1 or 2 splice sites initiation codon single or multiexon deletion) in a gene where LOF is a known mechanism of disease.

From ex vivo collagen phenotype analysis conducted on fibroblasts, it resulted that the discovered variant characterized by the insertion of six amino acids (Leu‐Ala‐Gly‐Pro‐Pro‐Gly) could alter the overall turnover of type III tropocollagen. This was evident by the marked reduction of its cellular excretion, likely derived from a reduced synthesis or an altered intracellular proteolysis process, as supported by the intracellular presence of low molecular weight collagen derivatives and lower levels of the type III collagen α1 chain (Figure 2).

Interestingly, the *in‐frame* duplication of the *COL3A1* gene is positioned 9 amino acids downstream from the canonical **Gly|Ile**‐Ala cleavage site of MMPs within a region rich in Ile and Leu residues (Figure 3).

It is worth noting that in type III collagen α1 chain, the 67% and 94% of Ile and Leu residues are located in the Xaa position, forming Gly‐Ile‐Yaa or Gly‐Leu‐Yaa triplets, which are widely acknowledged cleavage sites of MMPs (Laronha and Caldeira 2020). However, among all these triplets, only one, positioned at 948–950, represents the true collagenase cleavage site (Gly‐Ala‐Pro‐Gly‐Pro‐Leu‐**Gly**|**Ile‐**Ala‐Gly‐Ile‐Thr‐Gly‐Ala‐Arg) characterized by a distinctive sequence pattern with two imino acid triplets upstream (Gly‐Ala‐Pro‐Gly‐Pro‐Leu) and two non‐imino acids downstream (Gly‐Ile‐Thr‐Gly‐Ala‐Arg) (Xiao et al. 2010). In this context, the downstream insertion observed in this study (Leu‐Ala‐Gly‐Pro‐Pro‐Gly) may introduce a further new potential cleavage site (**Gly|Leu**) altering the sequence pattern involved in the collagenase domain activity of MMPs. Indeed, studies focused on the activity of MMP‐1 on type III collagen unveiled that the substitution of imino acid prolines in the Yaa‐position downstream the cleavage site resulted in the loss of proteolytic cleavage activity without altering the affinity of MMP‐1 to collagen (Williams and Olsen 2009). Moreover, Nuclear Magnetic Resonance interaction studies pointed out that the Ile and Leu residues of the collagen III α1 chain constitute key amino acids for the adhesion of the hemopexin domain of MMP‐1 to collagen (Bertini et al. 2012). These interactions are responsible for the local unwinding and destabilization of the triple helix, facilitating access of the single chain of collagen to the enzyme's active site (Karabencheva‐Christova et al. 2018). Taking all these findings into account, the observed Leu‐Ala‐Gly‐Pro‐Pro‐Gly duplication not only generates an additional potentially cleavable **Gly|Leu**‐Ala triplet but also introduces a hydrophobic Leu residue, which could destabilize the collagen structure, facilitating the direct collagenolytic activity of MMPs.

To test the potential structural alteration at the MMP1 cleavage site introduced by the variant, we performed a thermodynamic stability *in silico* study of the eight possible homo‐ and heterotrimers composed of the wild type and mutated chains. The results of such dynamic simulations of the triple helix structures showed a substantial analogous thermodynamic stability of the wild type and mutated, both of which have conserved the Gly‐Xaa‐Yaa triplets and hydroxyproline patterns crucial for the thermodynamic stability of the triple helix. However, a significant difference was found in the higher flexibility and curvature of the mutated protein compared to the wild type. The overlap of the mutated region with the binding region to the hemopexin domain of MMP‐1 (Figure 4) suggests that this different flexibility and adaptability of the collagen helix could facilitate the binding and unfolding of MMP to collagen III. Recent observations using Atomic Force Microscopy have revealed that the area of greatest flexibility along type III collagen is located precisely near the MMP cleavage site (Al‐Shaer et al. 2021). Considering the importance of partial unfolding in the mechanism of action of proteases in imino‐poor regions, the observed different dynamic behavior of the mutated chains compared to wild type may suggest an enhanced proteolytic activity of MMP1 towards the mutated collagen in agreement with the ex vivo experimental observations.

In conclusion, the results from both ex vivo experiment and the structural *in silico* simulation all together pointed out that the new c.2868_2885dup variant of the *COL3A1* gene could affect the overall turnover of collagen III, likely influencing the interactions with other collagen fibrils and extracellular matrix proteins, able to determine deep alterations in the tissue structural organization of the vessels and affect their relative stretch. The experimental findings of this study strongly suggest that the identified duplication can be considered a novel pathogenic variant of the *COL3A1* gene underpinning the clinical features suggestive of vEDS revealed in this family.

## Author Contributions

G.M., S.G., and G.C. contributed to the conceptualization and coordinated the study. P.M. performed and interpreted the histological and immunohistochemical data. S.V. performed and interpreted the fibroblast collagen analysis. G.M. and L.D.P. interpreted the molecular genetic analysis. S.G., M.D, and S.F. provided critical guidance in the structural protein characterization. G.M. collected and analyzed the clinical data. G.M., SG, and G.C. drafted the manuscript. All authors reviewed, edited, and approved the final draft.

## Conflicts of Interest

The authors declare no conflicts of interest.

## Supporting information


**Video S1.** Cartoon representation of 500 ns of molecular dynamics trajectory of the wild type chains (black/white‐grey/grey), with frames captured every 2.5 ns. The average flexibility (f) of this system is 0.00184 ± 0.00013 nm^−1^.

**Video S2.** Cartoon representation of 500 ns of molecular dynamics trajectory of the mutated chains (red/yellow/orange), with frames captured every 2.5 ns. The average flexibility (f) of this system is 0.00203 ± 0.00013 nm^−1^.

## Data Availability

The data that support the findings of this study are available on request from the corresponding author. The data are not publicly available due to privacy or ethical restrictions.
